# The Impact of Root Temperature on Photosynthesis and Isoprene Emission in Three Different Plant Species

**DOI:** 10.1100/2012/525827

**Published:** 2012-06-04

**Authors:** Mauro Medori, Lucia Michelini, Isabel Nogues, Francesco Loreto, Carlo Calfapietra

**Affiliations:** ^1^Institute of Agro-Environmental & Forest Biology (IBAF), National Research Council (CNR), Via Salaria km 29,300, 00015 Monterotondo Scalo, Rome, Italy; ^2^Department for Innovation in Biological, Agro-food and Forest systems (DIBAF), University of Tuscia, Via S. Camillo De Lellis snc, 01100 Viterbo, Italy; ^3^Department of Agronomy, Food, Natural Resources, Animals, Environment (DAFNAE), University of Padova, Agripolis, Viale dell'Università 16, 35020 Legnaro, Italy; ^4^Institute of Plant Protection (IPP), National Research Council (CNR), Via Madonna del Piano 10, 50019 Sesto Fiorentino, Florence, Italy

## Abstract

Most of the perennial plant species, particularly trees, emit volatile organic compounds (BVOCs) such as isoprene and monoterpenes, which in several cases have been demonstrated to protect against thermal shock and more generally against oxidative stress. In this paper, we show the response of three strong isoprene emitter species, namely, *Phragmites australis, Populus x euramericana*, and *Salix phylicifolia* exposed to artificial or natural warming of the root system in different conditions. This aspect has not been investigated so far while it is well known that warming the air around a plant stimulates considerably isoprene emission, as also shown in this paper. In the green house experiments where the warming corresponded with high stress conditions, as confirmed by higher activities of the main antioxidant enzymes, we found that isoprene uncoupled from photosynthesis at a certain stage of the warming treatment and that even when photosynthesis approached to zero isoprene emission was still ongoing. In the field experiment, in a typical cold-limited environment, warming did not affect isoprene emission whereas it increased significantly CO_2_ assimilation. Our findings suggest that the increase of isoprene could be a good marker of heat stress, whereas the decrease of isoprene a good marker of accelerated foliar senescence, two hypotheses that should be better investigated in the future.

## 1. Introduction

Isoprene is the most important biogenic volatile organic compound (BVOC) emitted by plants at terrestrial level. Isoprene emission accounts for about a third of total BVOC emission [1] and has well-known effects on plant biology and atmospheric chemistry. From the biological standpoint, isoprene emission allows plants to withstand higher temperatures and stronger oxidative pressure, probably because it strengthens membranes and quenches reactive oxidative species (ROS) [2]. In the atmosphere, isoprene may contribute to form ozone and particles, especially when reacting with anthropogenic pollutants [3].

Isoprene emission is controlled by environmental factors, mainly temperature and light availability [4]. Temperature has a dramatic impact on isoprene emission, which grows exponentially with temperature increase. This is mainly due to temperature-dependent activation of isoprene synthase [5] which, in the presence of adequate substrate availability, enhances synthesis of the volatile molecule. The Q_10_ of isoprene is 2–4 for air temperatures ranging between 25 and 35°C [4, 6]. Therefore, isoprene emission is expected to increase dramatically because of global warming, especially in temperate and boreal areas [7]. However, global warming is caused by the accumulation in the atmosphere of greenhouse gases, principally CO_2_. CO_2_ rise enhances photosynthesis but has a surprisingly negative effect on isoprene biosynthesis and emission [4, 8, 9]. This seems to be caused by inefficient competition of the isoprene biosynthetic pathway with respiratory processes for a common substrate, phosphoenolpyruvate (PEP) [8, 10, 11]. The increase of atmospheric CO_2_ may offset the temperature-driven stimulation of isoprene emission at global scale [12], but other models indicate that the impact of rising temperature might still lead to a higher isoprene load into the atmosphere, especially when some other indirect effects of rising CO_2_, such as the increase of biomass and leaf area density, are also considered [13].

Global warming will not only affect air temperature; it will also produce an increase of soil temperature which may have controversial consequences on soil ecosystems and soil organic matters. At the level of plant metabolism, the increase of temperature in the rhizosphere is expected to increase root respiration [14, 15]. However, the existence of acclimatory responses has been invoked to account for experimental observations about no or low respiratory increase under rising soil temperature [16].

The impact of rising root temperature on foliar emission of isoprene has, to our knowledge, never been studied to date. We hypothesize that increasing root temperature does not affect primarily isoprene emission at leaf level, unless heat propagates throughout the stem until leaf level. Such an indirect effect might be more easily observed in aquatic plants that have a developed aerenchyma to facilitate gas-exchange under anoxic conditions [17]. On the other hand, as rising temperature is expected to stimulate respiration of different organs of the plant, PEP may be preferentially allocated to respiration and isoprene emission may decrease because of substrate limitation. We carried out experiments to test the impact of rising root temperature on isoprene emission by three strong isoprene-emitting plants with laboratory and field experiments. A plant species that has large aerenchyma as adaptation mechanism to the anoxic conditions (*Phragmites australis*) and two typical hygrophylic trees (*Populus x euramericana *and *Salix phylicifolia*) were used.

## 2. Materials and Methods

### 2.1. Plant Material and Treatment Conditions

Experiments with common reed (*Phragmites australis* (Cav.) Trin. ex Steud) and hybrid poplar (*Populus x euramericana*) were carried out in a greenhouse of National Research Council (CNR) research area in Montelibretti (Rome) in 2009 and 2010, respectively. Inside the greenhouse, the air temperature was set at 25°C and the relative humidity at 60 ± 10% throughout the experiment. The mean photosynthetic photon flux density (PPFD) during measurements was ranging between 800 and 1000 *μ*mol m^−2^ s^−1^. Light was not supplied artificially and thus varied depending on meteorological conditions. Experiments were carried out during spring and summer, with daylight > 12 h over the entire period.

Rhizomes (each one having 3 buds) of *Phragmites australis* were grown in four pots containing each 10 L of tap water and agriperlite substrate until root and stem emission. The rate water/agriperlite was 4/1. In each pot, six individual rhizomes were placed. The water was oxygenated pumping air at a rate of 5 L min^−1^ into the pots. After leaf emersion, a 1/4 strength Hoagland's nutrient solution [18] was supplemented to tap water. The nutrient solution was subsequently made more concentrated to nourish a rapidly increasing biomass. At the end of this 45 d-long preliminary phase (i.e., at day 0 of true experiment), a full-strength Hoagland's nutrient solution, pH 7.6, was used. Two groups were then formed; two pots (hereafter named control treatment) were grown for other two months maintaining the hydroponic solution at 25°C, while the other two pots (warming treatment) were grown with the root system into water at a constant temperature of 40°C. The solution temperature was increased circulating the water into a heated water bath before reaching the pots. The temperature of the solution inside the pots was monitored continuously with a thermometer.

Shoot cuttings of similar size of hybrid poplar (*Populus x euramericana*) were also grown using a hydroponic system in 4 pots containing each 10 L of Hoagland's nutrient solution. In each pot, 8 cuttings were placed which were immediately exposed to control or warmed water. In particular, two pots were maintained at 27°C (control treatment), while in the other two pots, the temperature of the solution was increased to 38°C (warming treatment) by circulating the water into a heated water bath before reaching the pot.

As for common reed, in each pot, air was insufflated at a rate of 5 L min^−1^ to prevent anoxia of the root system.

Finally, field-grown *Salix phylicifolia* plants naturally growing in a hot spring situated in Hveragerdi Thermal Park (Iceland) were measured. During the field experiment, the mean air temperature was 10°C and maximum photosynthetic photon flux density (PPFD) ranged between 500 and 1200 *μ*mol m^−2^ s^−1^. Five plants growing inside the hot spring, whose roots were submerged by water at a temperature of 54°C, were compared with four plants growing nearby the park with an average temperature of the pond of 18°C during the measurements (June 2010).

### 2.2. Gas Exchange Measurements

In the laboratory experiments with *Phragmites australis*, gas exchange measurements were carried out starting on day 9 from the beginning of the warming treatment (i.e., when the first mature leaves were available) and subsequently 20, 27, and 34 days after starting the warming treatment. In the laboratory experiment with *Populus x euramericana*, five measurements were carried out between the end of May and August, covering a 72-d long period starting on day 30 (i.e., when the first mature leaves were available) and subsequently 38, 45, 75, and 102 days after the beginning of the warming treatment. In the field experiment with *Salix phylicifolia,* only measurements were carried out in one day, on plants permanently grown under different root temperature regimes. In all cases, only mature, fully expanded leaves in which isoprene emission is expected to be maximal [19] were selected for measurements. Measurements were replicated in four different plants of common reed and willow, and in five plants of poplar. One leaf per plant was measured.

The leaf was clamped in a 6 cm^2^ gas exchange cuvette and exposed to a 500 mL min^−1^ flow of synthetic air made by mixing 80% N_2_, 20% O_2_, and 400 ppm CO_2_. The cylinders of the three gases were of pure grade and the synthetic air did not contain trace gases, mainly hydrocarbons, that could have disturbed the measurement of isoprene. A portable infrared gas analyzer (LI-6400; Li-Cor, Lincoln, NE, USA) was used to determine CO_2_ and H_2_O exchange (photosynthesis, stomatal conductance, transpiration, and intercellular CO_2_ concentration) and to control the environmental factors. Measurements were carried out under a PPFD of 1000 *μ*mol m^−2^ s^−1^, at a leaf temperature of 30°C, and at a relative humidity between 50 and 60%. These are the conditions at which basal emission of isoprene is generally recorded [20]. In phragmites leaves, the measurements were repeated changing the air temperature from 20 to 35°C. To collect isoprene emission, the outlet of the leaf cuvette was connected to a tube filled with 200 mg Tenax. A pump was used to draw through the tube 5 L of the air flowing over the leaf inside the cuvette, at a rate of 200 mL min^−1^. Trapped compounds were immediately thermally desorbed at 275°C for 10 min and cryofocused in a cold trap for 3 minutes at −10°C. The cold trap was then flash heated at 300°C and the isoprene was injected trough a transfer line in a MS-5HP column with a internal diameter (id) of 0.25 mm (J&W Scientific USA, Agilent Technologies). The column temperature was held first at 40°C for 1 min, increasing to 210°C at a rate of 5°C/min and rising to a final temperature of 250°C at a rate of 20°C/min. The carrier gas was helium with constant pressure. The samples were analyzed by Gas Chromatography Mass Spectrometry (GCMS-MSD 5975C, Agilent). Isoprene emission was measured by comparing sample peak with peaks from a gaseous standard (100 ppb, RIVOIRA SPA, Chivasso, TO, Italy).

### 2.3. Biochemical Analyses

Biochemical analyses were carried out on *Populus x euramericana *samples, comparing control and warming samples (12 replicates each) after 50 days of treatment. Samples were cut and immediately frozen with liquid nitrogen. Reduced ascorbate (ASC), dehydroascorbate (DHA), as well as total and oxidized glutathione (GSSG), and the activity of the antioxidant enzyme catalase (CAT), ascorbate peroxidase (APX), and glutathione reductase (GR) were measured.

For preparing the leaf extracts used in the determination of ASC, DHA, total glutathione, and GSSG, the leaf tissue (about 100 mg fresh weight) was dissolved in 1.5 mL 3% perchloric acid and the mixture was centrifuged (5000 rpm, for 20 min) at 4°C. The pH was adjusted to 7 by adding 300–400 *μ*L of a sodium carbonate solution. ASC/DHA content was determined using the spectrophotometer method described previously [21]. For ASC, initial absorbance of a 50 *μ*L aliquot of extract was measured at 265 nm in 100 mM K-phosphate buffer (pH 6.1), then measured again 1 min after the addition of ascorbate oxidase (1 U mL^−1^). DHA content was determined in another 50 *μ*L aliquot. Initial absorbance was recorded as for ASC, and then the sample was measured again following the addition of 2 mM DL-dithiothreitol (DTT). An extinction coefficient of 14 mM^−1^ cm^−1^ for ASC at 265 nm was used in calculations [22]. Total glutathione was determined enzymatically. The reaction mixture contained: 100 mM phosphate buffer (pH 7.4), 5 mM EDTA, 1 mM DTNB, 0.5 mM NADPH, and 0.05 mL of leaf extract solution. After equilibration for 3 minutes at 25°C, the reaction was started by adding 2 units of glutathione reductase. The formation of 2-nitro-5-thiobenzoic acid was continuously recorded at 412 nm with a UV-vis spectrophotometer [23]. The total amount of glutathione in the samples was determined from a standard curve obtained by plotting the known amount of GSH versus the rate of change of absorbance at 412 nm. Samples for GSSG determination were incubated at room temperature with 20 *μ*L of 4-vinyl pyridine per 1000 *μ*L sample for 1 h. Incubation with 4-vinyl pyridine conjugates any GSH present in the sample so that only GSSG is recycled to GSH without interference by GSH.

For the measurements of enzyme activities leaf tissues were homogenized with 0.1 M phosphate buffer pH 7.8 in a prechilled mortar. The homogenate was centrifuged at 4°C for 20 min at 5000 rpm. Ascorbate peroxidase (APX) activity was determined spectrophotometrically by a decrease in absorbance of ASC at 265 nm (*ε* = 14 mM cm^−1^) [22]. The reaction mixture contained 50 mM potassium phosphate buffer pH 7, 5 mM ascorbic acid, 0.5 mM H_2_O_2_, and enzyme extract. Addition of H_2_O_2_ started the reaction. Activity was expressed as *μ*mol ASC min^−1^ mg protein^−1^. CAT activity was determined by consumption of H_2_O_2_ [24]. The reaction mixture contained 50 mM potassium phosphate buffer pH 7, 15 mM H_2_O_2_, and 20 *μ*L of enzyme extract. The consumption of H_2_O_2_ was monitored spectrophotometrically at 240 nm (*ε* = 0.0435 mM cm^−1^). The activity was expressed as *μ*mol H_2_O_2_ min^−1^ mg^−1^ protein. Gluthatione reductase (GR) was assayed by monitoring the glutathione-dependent oxidation of NADPH at 340 nm [23]. The reaction mixture contained 50 mM potassium phosphate buffer pH 7.4, 1 mM EDTA, 0.2 mM NADPH, and 50 *μ*L of enzyme extract. Reaction was initiated by adding 0.1 mL of 100 mM GSSG (oxidised glutathione). Protein concentrations were determined spectrophotometrically using Coomassie brilliant blue R-250 [25]. All assays were performed at 25°C.

### 2.4. Statistical Analyses

Differences between control and warming treatment were compared with *t*-test. All analyses were conducted with SigmaPlot 10.0 (Systat Software, Inc.). Differences were considered significant at level *P ≤ 0.05, **P ≤ 0.01, ***P ≤ 0.001.

## 3. Results

### 3.1. *Phragmites australis*

**Figure 1:**
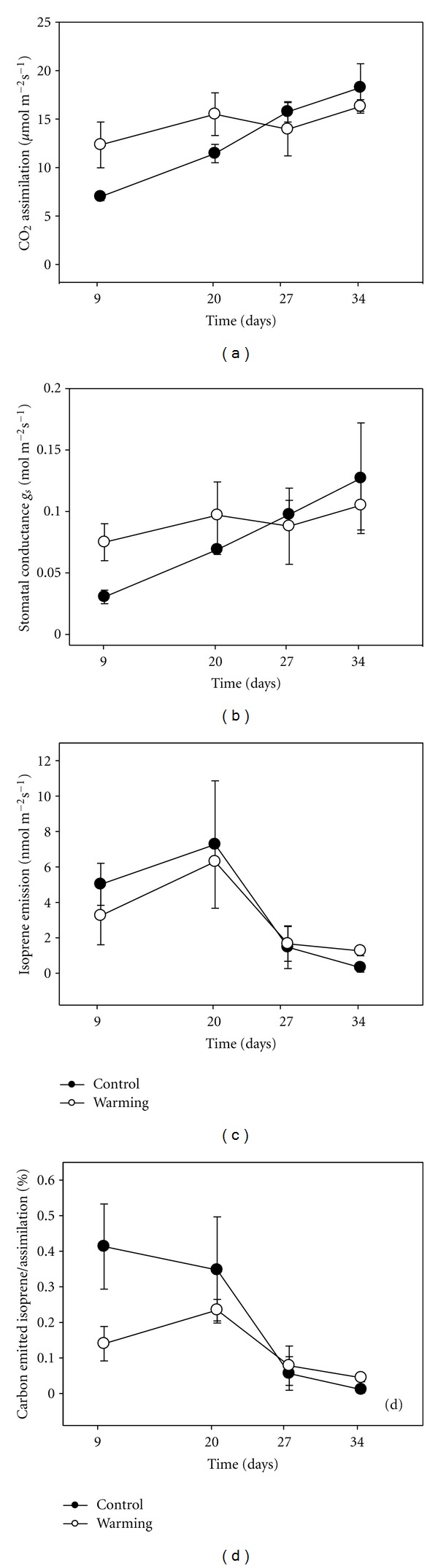
Time course of (a) CO_2_ Assimilation (*μ*mol m^−2^ s^−1^), (b) Stomatal conductance (mol m^−2^ s^−1^), (c) Isoprene emission (nmol m^−2^ s^−1^), (d) percentage of assimilated carbon lost as isoprene (%), ±S.E. for *Phragmites australis* grown in a hydroponic system with water at different temperatures at 9, 14, 21, and 27 days after start of treatment. Measurements started after 9 days from the beginning of the warming treatment, that is, as soon as the first mature leaves were available.

Net Photosynthesis (A) and stomatal conductance (gs) of plants in the warming treatment was higher than in control plants in the first part of the experiment (Figures 1(a) and 1(b)). However, due to the low number of replicates differences were not statistically significant. In control plants, A increased throughout the experiment up to a value around 18 *μ*mol m^−2^ s^−1^, while in the warming treatment, A and gs remained quite constant throughout the experiment. Isoprene emission was not different between the two treatments at the beginning of the experimental period (Figure 1(c)) and dropped in both treatments after 20 days. The percent of photosynthetic carbon reemitted as isoprene was higher in control than in the warming treatment at the beginning of the experiment, but then dropped in both cases to a similarly low value by the end of the experiment (Figure 1(d)).

**Figure 2:**
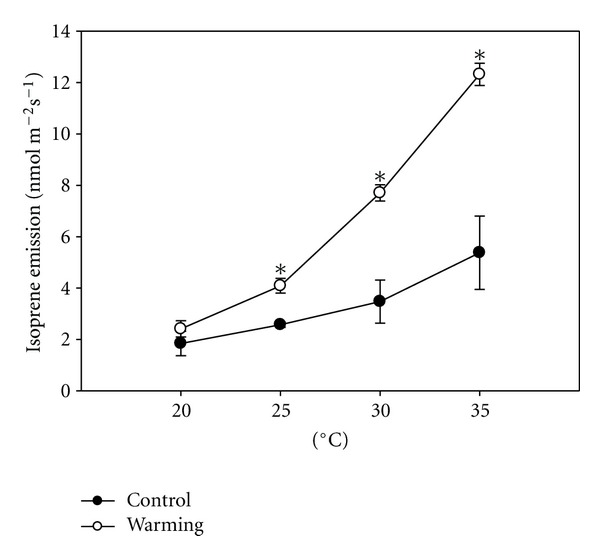
Relationship between isoprene emission rate and leaf temperature, ± S.E of *Phragmites australis* grown in a hydroponic system with water at different temperatures. *P ≤ 0.05, **P ≤ 0.01, ***P ≤ 0.001.

Figure 2 shows that increasing the air temperature inside the cuvette from 20°C to 35°C induced a stimulation in isoprene emission. This stimulation was significantly higher in the warming treatment than in control plants, in fact at the highest temperature (35°C), isoprene emitted by plants of the warming treatment was more than double than in control plants.

### 3.2. * Populus x euramericana*

**Figure 3:**
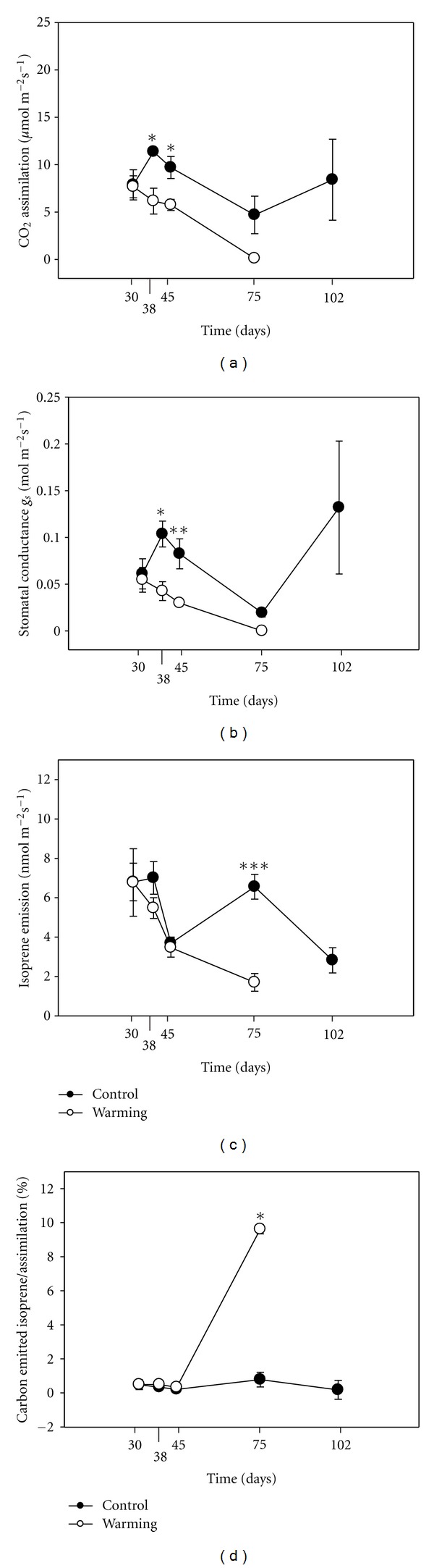
Time course of (a) CO_2_ Assimilation (*μ*mol m^−2^ s^−1^), (b) Stomatal conductance (mol m^−2^ s^−1^), (c) Isoprene emission (nmol m^−2^ s^−1^), (d) percentage of assimilated carbon lost as isoprene (%), ±S.E. for *Populus x euramericana* grown in a hydroponic system with water at different temperatures at 30, 38, 45, 75, and 102 days after start of treatment. *P ≤ 0.05, **P ≤ 0.01, ***P ≤ 0.001. Measurements started after 30 days from the beginning of the warming treatment, that is, as soon as the first mature leaves were available.

Gas exchange measurements on *Populus x euramericana* were taken starting 30 d after the beginning of the warming treatment. After the initial date of measurement, comparable A, gs, and isoprene emission were found between the warming and the control treatments (Figure 3). Subsequent measurements showed a strongly negative effect of the warming treatment on A and gs. A transient inhibition of A and gs also occurred in control plants 75 d after treatment, but in the warming treatment the effect was stronger, leading to the death of all plants in August. Isoprene emission also decreased during the warming treatment, whereas in control plants, the emission of isoprene after 75 d from the beginning of the treatment was as high as at the beginning of the experiment. Because of the resilience of isoprene emission, a much larger amount of photosynthetic carbon was allocated into isoprene by the end of the experiment, in control leaves.

**Figure 4:**
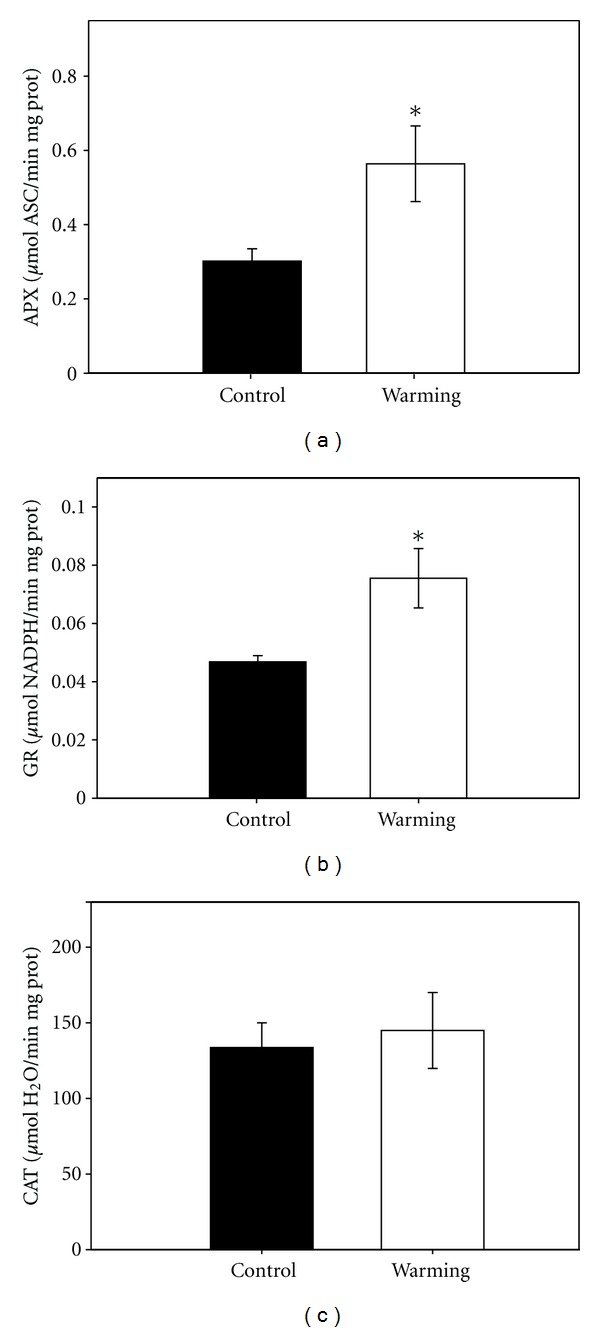
Activities of antioxidant enzymes related to the leaf protein content: (a) ascorbate peroxidase, (b) glutathione reductase, and (c) catalase (all expressed as *μ*mol min^−1^ mg^−1^ protein) in poplar control leaves and in leaves exposed to “warming.” Mean ± SE is shown for *Populus x euramericana* grown in a hydroponic system with water at different temperatures at 50 days after start of treatment. Differences between means of control and “warming”—treated leaves are shown. *P ≤ 0.05, **P ≤ 0.01, ***P ≤ 0.001.

The warming treatment also caused a general increase in the antioxidant enzymes (statistically significant in APX and GR, Figure 4) and in the antioxidant metabolites (Table 1) of poplar leaves, as assessed after 50 d of experiment. In particular, the warming treatment increased the total ASC pool by 25% and the total GSH pool by 60%. The increase of the ratio ASC/DHA in plants exposed to the warming treatment was particularly significant when assessed statistically (*P* < 0.0001), reflecting also a decrease of DHA pool in these plants. 

### 3.3. *Salix phylicifolia*

**Figure 5:**
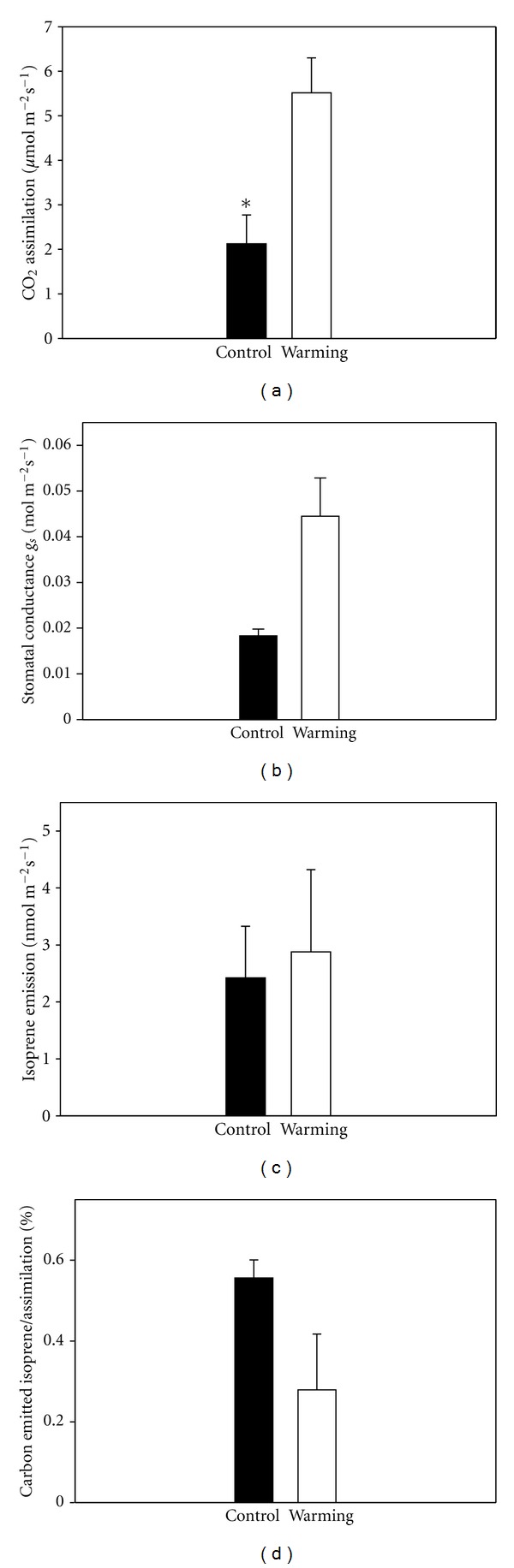
Time course of (a) CO_2_ Assimilation (*μ*mol m^−2^ s^−1^), (b) Stomatal conductance (mol m^−2^ s^−1^), (c) Isoprene emission (nmol m^−2^ s^−1^), (d) percentage of assimilated carbon lost as isoprene (%), ±S.E. for *Salix phylicifolia* grown close to the hot-spring (warming) and far away the hot water (control). *P ≤ 0.05, **P ≤ 0.01, ***P ≤ 0.001.

Photosynthesis and gs of *Salix phylicifolia *plants that were grown close to a natural hot spring under a naturally warmer root environment were higher than in plants growing at lower root temperature (Figures 5(a) and 5(b)). However, the difference was not statistically significant for gs (*P* = 0.10). 

The emission of isoprene was not affected by the warming treatment (Figure 5(c)). Consequently, the amount of photosynthetic carbon that was lost as isoprene was lower in the warming treatment than in control plants (Figure 5(d)).

## 4. Discussion

It is well known that isoprene emission is regulated by several environmental variables and that temperature is probably the most important [4]. However, in most studies, this has been observed under changing air conditions, while the effect of changing temperature in soil or soil water has never been investigated.

Isoprene emission rates of *P. australis *were similarly affected over the experimental period in control and in the warming treatment. The slight increase at the beginning of the experiment and the following sharp decrease of isoprene emission, therefore, was not caused by root warming. The decrease of isoprene emission was not associated to inhibition of A. In fact, A remained steady in the warming treatment and even remarkably increased in control plants. Despite isoprene biosynthesis being prevalently from direct shunting of photosynthetic metabolites [2], it is not rare that isoprene and A become uncoupled because of environmental changes such as under super or sub-ambient CO_2_ [4, 11] or high temperatures [26–28].

Our data clearly indicate that altering the root temperature can dramatically change the dependence of isoprene emission on air temperature (Figure 2). The emission of isoprene is very sensitive to the temperature [29–32]. Similarly, our results indicate an upregulation of the capacity to produce and emit isoprene in response to higher temperature of the root environment.

Our experimental system allowed us to control the air temperature of the leaf inside the cuvette but we do not know whether the warming of the root system also increased the temperature of the aboveground biomass. This could be expected especially in a species with large aerenchyma like *P. australis.* In any case, it might be hypothesized that the increased temperature dependency of isoprene emission by leaves exposed to root warming indicate a general adaptation mechanism that helps plants coping with high temperatures. Indeed, it has been shown that isoprene can improve the thermotolerance at temperatures significantly higher than 40°C especially in plants growing in environments characterized by frequent temperature changes [33]. Isoprene probably plays a double role, stabilizing membranes and scavenging reactive oxygen species within the leaves [2]. In particular, when coping with heat stress, isoprene stabilizes thylakoid membranes improving the functionality of the photosynthetic complexes thereby embedded [26, 34, 35].

The differences between treatments showed in Figure 2 were not evidenced in Figure 1(c), probably because in the first case temperature curves were performed at a different development stage of plants or because we manipulated the air temperature around the leaf in the first case while just the water temperature and thus the root temperature in the second case.

On *P. x euramericana* plants, a 10°C increase of the water temperature in the root system reduced photosynthesis and stomatal conductance on average by 40%. This is a totally different effect than observed in the aquatic species *P. australis*. However, the effect was again noticed also in control plants, though at a later stage. Plants often show an onset of a premature senescence in response to warming stress [36]. The increase in antioxidant enzyme activities that we observed suggests that stressed plants had an effective system for detoxifying active oxygen species. On the other hand, the warming treatment may have affected the normal ontogeny of leaves, accelerating leaf development. On the basis of our results, it can be suggested that severe warming stress is responsible for premature senescence as indicated by the increase of enzyme activities (Figure 4) and has caused irreversible inhibition of photosynthesis (Figure 3). Significant increases in the activities of APX during the late stage of leaf development have been already described in sweetpotato leaves [37]. In our case, the increase in APX and CAT activities may be also related to senescence. This would be in agreement with the decrease in photosynthesis typical of leaf senescence and that can be observed in hot water treated plants until July.

Elevated root temperature could have simply anticipated a possibly age-related limitation of photosynthesis, simply by insufficient carbon acquisition through stomatal closure, or because of rapid decay of Rubisco properties. However, whereas all plants grown in hot water died 75 d after starting the treatments, control plants were able to survive along the experimental period and in fact recovered the original rates of A and gs by the end of treatment. Interestingly, recovery of photosynthetic properties was preceded by a significant increase of isoprene emission in control leaves, perhaps indicating the activation of molecular mechanisms related to general defense against environmental stress, or more likely scavenging reactive oxygen and nitrogen species [38, 39].

Hartikainen et al. [40] showed that isoprene emission tended to drop under elevated temperature in one genotype of aspen whereas the second genotype was able to sustain higher isoprene level under warming treatment. In other studies, isoprene was inhibited by very high temperature, but under the recovery period, both isoprene emission and photosynthesis turned back to control values [28, 41]. This was assumed to indicate denaturation of isoprene synthase [41], but Loreto et al. [28] challenged this view, suggesting instead that isoprene could be temporarily inhibited by substrate limitation. Since the reduction of isoprene emission was associated with photosynthesis inhibition on both control and warming treatment during the first days of the experiments, photosynthesis limitations may be a more likely explanation for the temporary reduction of isoprene. It is known that alternative sources of carbon can be activated for isoprene biosynthesis under stress conditions that make photosynthesis an inefficient source [42–44]. The activation of these sources might have occurred under the warming treatment, therefore, explaining the burst of isoprene 75 d after this treatment.

From the data collected in Iceland, it is evident that* Salix phylicifolia *plants grown close to the hot springs are well adapted to the extreme conditions represented by high temperature. In fact, the proximity to the hot springs seems to have produced a benefit in terms of higher assimilation rates. This might be due to both warmer temperature, in an environment typically limited by low temperatures, and by possible higher levels of CO_2_. We might hypothesize the steam was rich not only of water vapor but also of CO_2_ which might have caused a stimulation of CO_2_ uptake. Our results agree with McLeod et al. [45], who also showed higher values both for photosynthesis rates and stomatal conductance for *Salix nigra* plants treated with 40°C hot water. 

It is interesting to remark that isoprene emission was not stimulated by the proximity with the hot spring.

This uncoupling between isoprene emission and photosynthesis may be due to long-term acclimation of isoprene biosynthesis to growth conditions, or to the absence of stress conditions that would stimulate isoprene biosynthesis [39]. However, it cannot be excluded that a more robust experimental dataset would reveal that a slight increase of isoprene emission also occurs under this condition.

In conclusion, our experiments reveal that root warming does not lead to unequivocal changes of isoprene emissions, and of photosynthetic parameters. In the three scrutinized cases, we found that isoprene uncoupled from photosynthesis at a certain stage of the warming treatment. We take the increase of isoprene as a marker of heat stress, and the decrease of isoprene as a marker of accelerated foliar senescence, two hypotheses that should be more accurately tested with the help of biochemical and genetic markers in the future.

## Figures and Tables

**Table 1:** Contents of the ascorbate and glutathione related to the fresh weight: reduced ascorbate (ASC), oxidised ascorbate (DHA), total ascorbate (all expressed as *μ*mol g^−1^ f.w.), and ascorbate ratio (ASC/DHA); reduced glutathione (GSH), oxidised glutathione (GSSG), total glutathione (all expressed as *μ*mol g^−1^ f.w.), and glutathione ratio (GSH/GSSG) in poplar leaves under control and warming treatments. Mean ± SE is shown. Differences between means of control and treated leaves are shown with the *P* value. Significant value is with *P* < 0.05. The activities of antioxidant enzymes were related to the leaf protein content whereas the ascorbate and glutathione contents were related to the fresh weight.

	Control	Warming	*P* value
ASC	7.55 ± 0.38	10.01 ± 0.65	**0.004**
DHA	2.82 ± 0.43	2.76 ± 0.19	0.678
Total ASC	10.36 ± 0.57	12.76 ± 0.68	**0.0145**
ASC/DHA Ratio	2.68 ± 0.16	3.63 ± 0.09	**0.0001**
GSH	0.11 ± 0.03	0.22 ± 0.05	0.0823
GSSG	0.18 ± 0.02	0.25 ± 0.02	**0.011**
Total GSH	0.29 ± 0.02	0.47 ± 0.04	**0.002**
GSH/GSSG Ratio	0.64 ± 0.28	0.86 ± 0.24	0.5452
